# Inflammation and Vasculitis Related to Brolucizumab

**DOI:** 10.3390/jcm13175208

**Published:** 2024-09-02

**Authors:** António Campos, Carolina Mota, Francisco Caramelo, Nuno Oliveira, Sara Silva, João Sousa

**Affiliations:** 1 Ophtalmology Department, Centro Hospitalar de Leiria, 2410-197 Leiria, Portugal; carolina.mota@ulsrl.min-saude.pt (C.M.); nuno.oliveira@ulsrl.min-saude.pt (N.O.); sara.pereira@ulsrl.min-saude.pt (S.S.); joao.sousa@ulsrl.min-saude.pt (J.S.); 2 ciTechCare, Center for Innovative Care and Health Technology, Polytechnic Institute of Leiria, 2414-016 Leiria, Portugal; 3 Center for Innovative Biomedicine and Biotechnology (CIBB), University of Coimbra, 3004-504 Coimbra, Portugal; fcaramelo@fmed.uc.pt; 4 Sciences Department, Faculty of Health Sciences, University of Beira Interior, 6201-001 Covilhã, Portugal

**Keywords:** anti-VEGF, brolucizumab, age-related macular degeneration, neovascular age-related macular degeneration, retinal vasculitis, retinal artery occlusion, intra-ocular injections, inflammation, endophthalmitis

## Abstract

**Background/objectives**: To compare the prevalence of intra-ocular inflammation (IOI) between brolucizumab and aflibercept in neovascular age-related macular degeneration (nAMD) after intra-vitreal injections (IVI) and to compare the IOI odds ratios (ORs) of both therapies with the prevalence of septic endophthalmitis after IVI that was previously reported in the literature. **Methods**: A total of 468 IVI of brolucizumab (117 eyes) were compared with 2884 IVI of aflibercept (305 eyes) regarding IOI and occlusive retinal vasculitis (RV) from December 2021 to June 2023 in this retrospective study. The OR was calculated for both anti-VEGF agents and was compared with the relative risk of septic endophthalmitis after IVI. **Results**: There were four eyes with unilateral IOI related to brolucizumab (3.42%), one presenting uveitis (0.85%), two vitritis (1.71%) and the last one presenting occlusive RV (0.85%), compared with two eyes presenting unilateral IOI (anterior uveitis, 0.66%) and none with RV from the aflibercept cohort. The incidence of IOI per injection with brolucizumab (0.855%) was significantly higher compared with aflibercept (0.069%, *p* = 0.004). The OR of IOI related to brolucizumab IVI compared with septic endophthalmitis was 20 times greater (1.49 for aflibercept, *p* = 0.646, versus 20.15 for brolucizumab, *p* < 0.001). The OR of RV with brolucizumab compared with septic endophthalmitis was 4.6. **Conclusion**: Data from our department suggest a much higher risk of IOI and occlusive retinal vasculitis after brolucizumab when compared with aflibercept. The risk of IOI and severe sight-threatening complications related to brolucizumab is greater than the risk of septic endophthalmitis after any IVI.

## 1. Introduction

Brolucizumab is a humanized monoclonal antibody single-chain variable fragment, which proficiently inhibits all variants of VEGF-A [1]. Its desirable attributes, such as its small molecular weight and excellent solubility, have rendered it immensely attractive to ophthalmologists, since these qualities allow the administration of higher doses at longer intervals between intra-vitreal injections (IVI) [2]. It was approved on 7 October 2019, by the US Food and Drug Administration (FDA) at a dosage of 6 mg for the treatment of neovascular age-related macular degeneration (nAMD) [3]. The phase 3 HAWK and HARRIER trials demonstrated that 8- (q8) or 12-week (q12) intervals of administration of brolucizumab are non-inferior to every other month aflibercept (q8) [4]. The ability to space the IVI and the good efficacy in drying the macula are related to its nearly 12-time equivalent molar dose when compared to aflibercept, providing therefore, a greater half-time when administered into the vitreous [5,6]. Spacing IVI when treating nAMD without recurrence is an aim any department of ophthalmology strives for, considering that the control of the disease in the long-term is related to the number of injections, and under-treatment with some degree of vision loss has been reported as a worrisome shortcoming [7,8,9].

The most commonly reported adverse reactions found in the aforementioned trials were reduced visual acuity (7.3%), cataract (7.0%), conjunctival hemorrhage (6.3%), and vitreous floaters (5.1%). The most serious adverse reactions included blindness (0.8%), endophthalmitis (0.7%), retinal vasculitis (RV) and retinal arterial occlusion (RO) (0.8%), and retinal detachment (0.7%) [10]. However, post-marketing studies presented in February 2020 by the American Society of Retinal Specialists (ASRS) reported 14 cases of RV, 11 of which were associated with RO [11]. In May 2020, the first wake-up call on brolucizumab’s serious side effects was published in the form of an editorial [12]. Since then, numerous case reports, case series, retrospective studies, and a recent systematic review have been published, all documenting the link between brolucizumab and occlusive RV associated with intraocular inflammation (IOI). Some of these studies have reported cases resulting in severe and irreversible vision loss [2,13,14]. The prevalence of IOI and RV or RO reported in a post hoc analysis of the HARRIER and HAWK trials conducted by the Safety Review Committee (SRC), an independent committee commissioned by Novartis, was 4.6% and 2.1%, respectively [4,15]. This analysis reported that the RO risk rate for all-brolucizumab-treated patients was low (0.46%). However, due to the devastating nature of this complication and the high number of patients on treatment for nAMD, from the early days, several authors claimed that brolucizumab therapy should be closely monitored or suspended [12,16].

Data from the Merlin study increased these concerns and, as a consequence, the retinal vein occlusion trials RAPTOR and RAVEN were halted by Novartis [17]. Moreover, a recent multicenter trial enrolling 1351 eyes in Japan found incidences of IOI of 11.3%, RV and RO of 3.9% and severe or total visual loss of 1.9% [18]. To understand and weigh the importance of these data, we thought it might be useful to compare them with the incidence of another devastating consequence of treating nAMD with IVI which is independent of the drug administered, that is, septic endophthalmitis. Septic endophthalmitis is a fearful consequence of IVI; hence, it has been a recurrent and permanent concern of the scientific community because severe visual loss or blindness may involve one-fourth to one-third of the eyes due to this complication [19,20,21,22,23,24,25]. A cumulative incidence of endophthalmitis after IVI of 0.048% has been reported [26,27,28]. Therefore, to weigh up the IOI risk of using brolucizumab, we compared the relative risk per IVI between aflibercept and brolucizumab, and compared this with the previously reported risk of septic endophthalmitis per IVI, regardless of the drug injected.

## 2. Material and Methods

### 2.1. Eyes, Injections and Imaging

All eyes undergoing brolucizumab therapy for nAMD at the Leiria Hospital Centre from December 2021 to June 2023 were included in this retrospective, non-controlled, institutional study. To serve as a control group, all eyes treated with aflibercept for nAMD within the same period of time were included as well. The frequency and relative risk of IOI and RV/RO were calculated regarding the number of events per number of injections (and not per number of eyes) of either drug and compared with the risk of septic endophthalmitis per IVI previously reported in the literature. The exclusion criteria included other causes of macular edema such as diabetic retinopathy, retinal vein occlusion, macular telangiectasia or angioid streaks, and history of previous uveitis or concomitant systemic inflammatory conditions likely to result in misleading data. The data were collected from institutional clinical files from the National Registry platform of the Portuguese Retina Study Group (GER), Retina.pt. The collected data included age, gender, date of symptoms onset and duration until admittance to the hospital, elapsed time since the last injection, number of injection givens, laterality, clinical findings, nAMD subtype, best-corrected visual acuity (BCVA) at presentation and discharge, therapy for IOI and time to discharge. Each participant provided written informed consent. The platform Retina.pt was approved by the Ethics Committee of our hospital several years ago and by most of the Committees of the Portuguese hospitals, if not all. Optical coherence tomography (OCT) and fluorescein angiography (FA) were performed by certified site personnel using spectral domain optical coherence tomography (SD-OCT) and angiograph, Heidelberg GmBH, Germany. BCVA was collected using Early Treatment Diabetic Retinopathy Study Group (ETDRS) charts to avoid bias from non-logarithmic charts.

For safety reasons, the brolucizumab IVI protocol of our department was adapted from the Hawk and Harrier trials to have wider intervals [4], including a loading dose comprising 3 injections, each 6 weeks apart, for naïve patients, the same interval but not the same loading dose, as used in the KESTREL and KITE studies for diabetic macular edema [29]. However, eyes switching from 8-week aflibercept skipped the loading dose and were initiated into 8-week brolucizumab. There were no naïve eyes enrolled, that is, all eyes were switched from aflibercept. The study adhered to the principles of the Declaration of Helsinki.

### 2.2. Statistical Analyses

Statistical analyses were performed to examine the incidence and association of two conditions, A (aflibercept) and B (brolucizumab). First, a one-sample binomial test was conducted to compare the incidence of IOI in each condition (A and B) in our sample with the known incidence of infectious endophthamitis after IVI (0.048%) [26]. To investigate the possible association between conditions A and B, we applied Fisher’s exact test. Lastly, we performed a simulation to calculate the odds ratio (OR) associated with the conditions A and B for IOI and RO/RV, following a procedure our group published previously [28].

All statistical analyses were performed using R software (version 4.1.2), statistical platform R v4.1.2, the R Foundation Vienna, Austria, and a *p*-value < 0.05 was considered statistically significant.

## 3. Results

A total of 468 IVI of brolucizumab for nAMD (117 eyes) in our department, from December 2021 to the end of June 2023, were included. A total of 2884 IVI of aflibercept (305 eyes) to treat nAMD within the same period of time were included and were used as a control group. The aflibercept cohort had two cases of IOI and none of RV/RO within the aforementioned period of time. Regarding the brolucizumab cohort, there were four eyes (3.42%) with unilateral IOI: one presenting anterior uveitis (0.85%), two vitritis (1.71%), and one presenting RV with RO (0.85%) with dramatic vision loss. Regarding the number of IVI given, there were two cases of IOI with aflibercept per 2884 IVI (0.069%) and four cases of IOI with brolucizumab per 468 IVI (0.855%), a probability 20.15 times greater, *p* < 0.001 (Supplementary File S1).

### 3.1. Case Description

The first case of IOI occurred on May 2022, after the third IVI of brolucizumab, when 74 eyes (63.2%) were already undergoing brolucizumab therapy. The remaining three IOI cases occurred after the first (n = 2) and second (n = 1) IVI. The RV/RO case occurred after the second IVI and it was the latest and the last documented case of IOI (Table 1). There was no female to male predominance (two cases each). The age of cases ranged from 62 to 84, and three cases were treated for polypoidal choroidal vasculopathy. None of the cases were naïve on brolucizumab, and they were all switched from aflibercept, either with the goal of having a complete resolution of fluid or having wider administration intervals. The first brolucizumab IVI was given 6 to 9 weeks after the last injection of aflibercept. Signs and symptoms appeared 7 to 21 days after the last IVI of brolucizumab. They included floaters, keratic precipitates, a decrease in BCVA, vitritis, and in the case with RO, pain, redness, vision loss and ciliary congestion (Table 1). BCVA changed from +4 L to −72 L after the IOI event. All cases recovered BCVA completely up to pre-event levels, except for the case with arterial RO that was discharged with legal blindness (32 L, <20/200, −40 L). 

### 3.2. Case with Retinal Vasculitis and Retinal Arterial Occlusion

**Figure 1:**
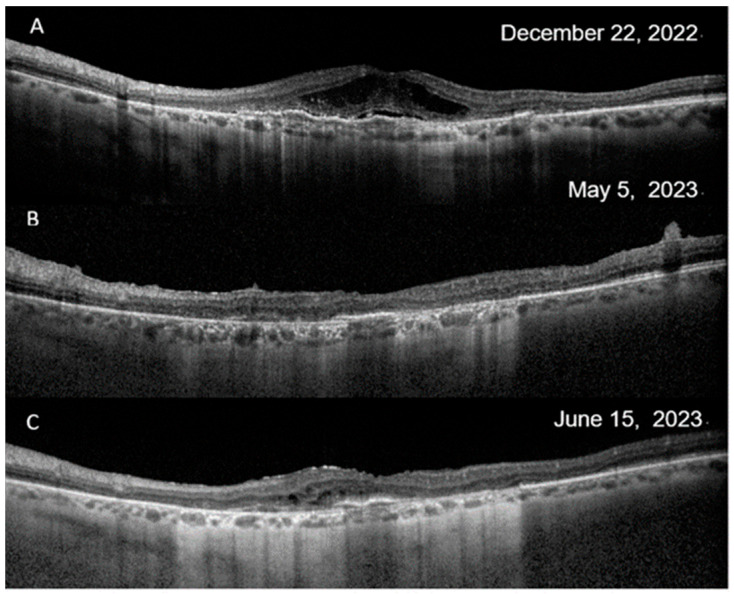
Optical coherence tomography (OCT) findings in the event with retinal artery vasculitis and artery occlusion. (**A**). On q8 aflibercept, there were subretinal fluid and intra-retinal cysts, despite a BCVA of 20/50 +2 L. (**B**). After the second injection of brolucizumab, there was no fluid in the retina, but there was edema of the inner retinal layers and diffuse atrophy of the retina. There was submacular scarring and extensive atrophy of the ellipsoid zone. BCVA dropped to counting fingers. (**C**). Before discharge, there was extensive atrophy and some retinal cysts before the patient resumed aflibercept. BCVA was < 20/200.

**Figure 2:**
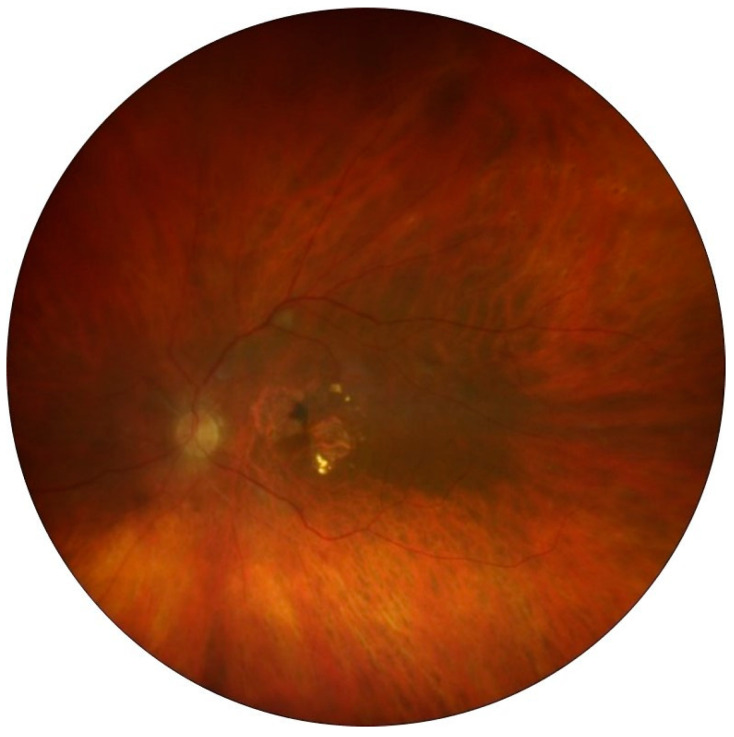
Color fundus photography (CFP). There were diffuse vascular sheathing involving arteries in the four quadrants, narrowing of the arterial and vein diameters, and cotton wool spots, but notably, retinal hemorrhages were absent.

**Figure 3:**
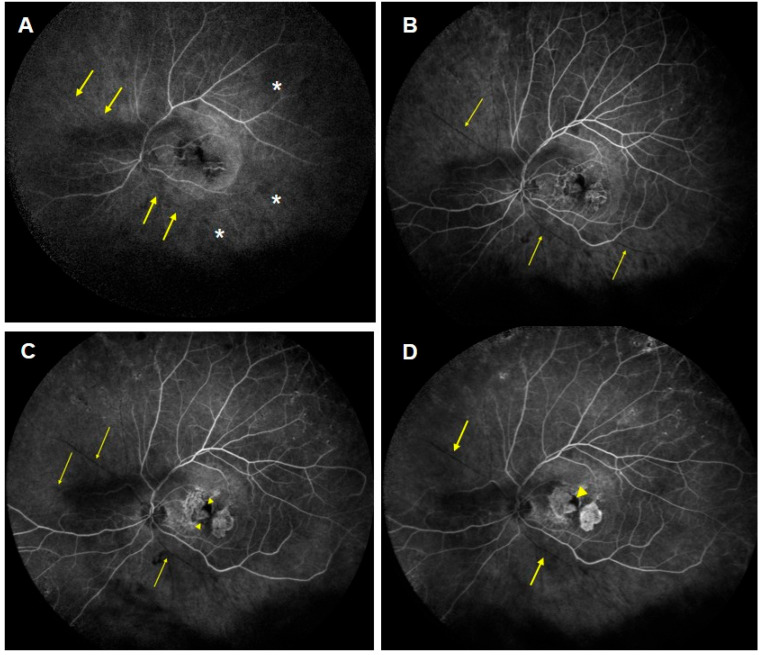
Fluorescein angiography. (**A**) (40″) and (**B**) (50″). Early choroidal multifocal hypofluorescence related to hypoperfusion (asterisks) and retinal arterial filing defects in the form of amputation of flux in the superior nasal and inferior temporal arteries (arrows), along with superior temporal and ciliary arteries’ delay. (**C**) (1′09″) and (**D**) (2′09″). Wide extensions of non-perfusion were present in the nasal and temporal areas, with venous narrowing and late filling as well as macular ischemia (arrowheads).

In particular, the case with RV and RO was a 70-year-old Caucasian man, reporting redness and pain in his left eye (LE) for the past eight weeks following his last IVI of brolucizumab. It was the second injection of brolucizumab, 8 weeks apart from the first one. The patient had a history of polypoidal choroidal vasculopathy in both eyes. Over the past three years, the patient consistently underwent intravitreal treatments, starting with ranibizumab and switching to aflibercept. The patient had a medical history of Type 2 diabetes and cardiac arrhythmia, which required a pacemaker. He underwent bilateral cataract surgery three years before. There was no history of IOI, known allergies or systemic inflammatory diseases. The patient’s LE showed a BCVA of 72 letters, along with some loss of the ellipsoid zone and elevation of the retinal pigment epithelial line, recurrent subretinal fluid and intraretinal cysts, despite being under aflibercept therapy (Figure 1A). He was switched from q8 aflibercept to q8 brolucizumab. After the first brolucizumab IVI, at the time of the second brolucizumab IVI, the subretinal fluid and cysts had resolved entirely. Following the second brolucizumab IVI, the patient experienced pain and redness in his LE for two months before he sought medical care. At the emergency visit, his LE’s BCVA dropped to counting fingers. The intraocular pressure was 25 mmHg, and clinical examination revealed conjunctival and ciliary hyperemia and 2+ anterior chamber cells. Fundus examination showed moderate vitritis and diffuse vascular sheathing involving arteries in the four quadrants and cotton wool spots, but notably the absence of retinal hemorrhages (Figure 2). There was hiperreflectivity of the inner retinal layers and diffuse retinal atrophy on OCT, indicating retinal ischemia (Figure 1B). On FA, there were early choroidal multifocal hypofluorescence related to hypoperfusion and retinal arterial filling defects in the form of amputation of flux in the superior nasal and inferior temporal arteries (Figure 3A), along with superior temporal and ciliary artery delays with venous late filling and macular ischemia. Wide extensions of non-perfusion were present in the nasal and temporal areas (Figure 3B–D). Treatment started with topical 1% dexamethasone, tropicamide and 64 mg oral methylprednisolone. A 700 μg dexamethasone intravitreal implant was administered four days later.

Three weeks later, BCVA was 30 L, and there was absence of flare in the anterior chamber. A subtle vitritis persisted, but there was a pale optic disc, peripapillary vascular sheathing, and inferior and nasal superior arterial segmentation. Oral and topical steroids were tapered, and due to disease relapse in the form of intra-retinal cysts, he was resumed on aflibercept (Figure 1C). Our department stopped using brolucizumab on June 2023.

### 3.3. Statistical Analyses and Relevance

To assess the seriousness of IOI related to brolucizumab, we compared IOI related to brolucizumab (condition B) and IOI related to aflibercept (condition A) with the previously reported incidence of infectious endophthalmitis after IVI of 0.00048 (Table 2). IOI following IVI of brolucizumab was statistically significantly higher than the incidence of septic endophthalmitis per IVI, unlike aflibercept (*p* < 0.0001 versus *p* = 0.403). The Fisher’s test confirmed that the direct differences in IOI between brolucizumab and aflibercept were also significant (*p* = 0.004).

The general incidence of endophthalmitis after IVI P(E), the probability of the event E to happen, is assumed to be 0.048%. The incidence for each of the anti-VEGFs (A and B) is the probability of the event E to happen under conditions A [P(E/A)] and B [P (E/B)], assuming that the conditions A, B, and C are independent.

To determine whether the proportion obtained with each of the conditions is different from the general incidence of the event, a binomial test of a sample was made for each condition using the number of cases where the event took place and the number of total cases, comparing thereafter with the general proportion of 0.00048. The statistical platform R v3.3.2 was used to find the P values. Brolucizumab use was related to a statistically significant increased risk of IOI.

We also calculated the ORs for conditions A and B (Table 3). The OR indicates that Brolucizumab has at least a 20.15-fold greater risk of causing IOI than any IVI has of causing septic endophthalmitis (*p* < 0.0001), while aflibercept has only a non-statistically significant 1.49 times greater risk (*p* = 0.646). 

It was calculated if there was an increased risk associated with conditions (drugs) A and B, taking into account the relative incidence of IOI for each drug found in the study under consideration and the general (expected) incidence of endophthalmitis, P (E). By varying the N, the odds ratio between the 2 conditions may be calculated when the number of IVI increases to infinite. The values for the odds ratio were calculated to increasing numbers of N above the minimum number obtained to respect a statistical significance of α = 0.05. Brolucizumab use was related with an increased risk of IOI. It is of note that the values of the odds ratio decrease with the increase of N, but the nature of such variation suggests the existence of an asymptote for which the true values of the odds ratio run to, given the initial conditions of the problem. To calculate the odds ratio, a general expression was obtained for condition A (aflibercept), from the probability of the occurrence of the event P(E):

$$OR=\frac{2\times \left[N\times \left[1-\left(E\right)\right]-2882\right]}{2882\times N\times \left(E\right)}$$

The same expression was then used to calculate the odds ratio for condition B, by adjusting the number of events for brolucizumab. Because the minimum value of a cell is 1, it was determined what would be the minimum value of N that would fit the conditions of the problem. As it is, the expression is applicable:

The huge number of injections needed to find and quantify evidence explains why prospective works are difficult to perform with rare occurrences.

If we consider RV/RO related to brolucizumab alone, there is a 4.99-fold greater risk than regular IVI of causing infectious endophthalmitis. All these calculations are available in Supplementary File S1.

## 4. Discussion

Brolucizumab is chosen to treat nAMD due to a molecular concentration 11 to 22 times greater than what can be achieved by aflibercept or ranibizumab [30], theoretically allowing wider time gaps between injections and superior drying of the retina [4]. Nevertheless, from the early days, several warnings about its deleterious side effects and reports on patients losing vision, led the ASRS to enforce the need to report such cases [11,12,30]. In this context, Novartis commissioned the SRC to study IOI events related to brolucizumab [31]. Later, the results of the MERLIN trial, in which the incidence of IOI, including RV and RO, were 9.3% (0.8% and 2.0%) for brolucizumab versus 4.5% (0% and 0%) for aflibercept, confirmed the potential toxicity of brolucizumab, discouraging its use at a 4-week interval [17].

The aim of this study was to report the prevalence of adverse events related to brolucizumab IVI in our department, with a specific focus on IOI and RV/RO, because serious side effects of brolucizumab need to be reported in order to provide robust data for analysis by the regulatory authorities. Although we understand that there may be anecdotal unreported cases, herein we actually report the first case of RV, RO and severe vision loss related to brolucizumab occurring in Portugal.

None of our patients had systemic inflammatory diseases, history of uveitis or retinal lesions that could mislead the diagnosis, and the patients improved with the use of steroids, as reported earlier by other authors [30]. We found a rate of 3.42% of IOI related to brolucizumab, slightly less than the 4.6% found in the Hawk and Harrier trials, and the rate of retinal artery occlusion was similar (0.85% versus 0.9%, respectively) [16]. This compares with a recently reported rate of 8.6% and 1.2%, respectively [32]. However, in the OCTOPUS and SWIFT trials, brolucizumab-related IOI events were up to 10.5% [33]. One might think that a 0.85% event rate of RO is a negligible incidence of a side effect. However, the seriousness and the dreadful consequences of an arterial occlusion may be compared with another serious complications of IVI, e.g., septic endophthalmitis. The actual incidence of septic endophthalmitis after IVI is 0.048%, which is an incidence 18 times less [26,28]. If we compare this with the incidence of any IOI of 19.1 per 1000 injections (1.9%), corresponding to an incidence of RO or RV of about 0.38%, this would still be an incidence 10 times greater than that for infectious endophthalmitis [33]. However, in the Intelligent Research in Sight (IRIS) registry and Komodo Health Care Map, the incidence of RV or RO was higher, up to 0.6% [34]. We also compared the incidence of IOI related to brolucizumab and to aflibercept by a Fisher’s test and found a strong, statistically significant difference in the risk of IOI associated with brolucizumab. The OR indicates that the risk of having an IOI with brolucizumab was 20.15 times greater than the risk of endophthalmitis associated with the IVI alone, and 4.99 times greater when considering RV/RO. Considering data from the manufacturer, Novartis AG, on its site, reported an incidence of RV/RO of 13.8/10.000 IVI, which is 0.14% or 2.92 times the incidence of infectious endophthalmitis [18]. A recent multicenter retrospective study in Japan reported an incidence of IOI of 11.3% (versus 3.42% in our report) and of RV/RO of 3.9% (versus 0.85% in our report), suggesting that the OR we found in our results may actually turn out to be underestimated [18].

According to Hawk and Harrier, brolucizumab is non-inferior to aflibercept, but taking into account the aforementioned numbers, it is arguable whether it is worth using brolucizumab just for an eventual gain in the treatment interval for the sake of comfort and reduced burden while ignoring the safety risk [4]. Moreover, aflibercept proved to be safer, with a rate of RO at least nine times less than that of brolucizumab (0.1% versus 0.9%, respectively) [35,36]. This is of overwhelming importance if we take into account recent reports indicating the need to be more proactive in the treatment of nAMD, probably with more injections than in the real-world current practice, to avoid under-treatment [8]. In addition, there are new therapeutic options that may achieve the same goal, perhaps without taking this level of risk, even though there is much to be known on this matter in the near future [37,38].

There was no female to male predominance in our occurrences, unlike in the reports of the ASRS and other series [2,14,34]. The fact that the first case occurred after more than 50% of eyes were already on treatment and after the third injection only warns against the false sense of security that we experienced and that other colleagues might have in the future as well. Of note, for safety reasons, we adapted the brolucizumab’s protocol for naïve patients, using larger intervals than were adopted in the Hawk and Harrier trials, even before the evidence brought by the MERLIN trial [4,17]. We included non-naïve eyes only, but skipped the loading dose protocol, adopting a q8 interval to start with. The onset of IOI events occurred early, within the first to the third brolucizumab injection and 7 to 21 days after the last injection (Table 1), which is in accordance with the OCTOPUS and SWIFT trials or in line with a recent review reporting 88% of cases within 30 days, after a mean of 1.7 brolucizumab injections before the event [2,33]. The time to discharge from 2 to 9 weeks, longer when RO was present, was slightly less than what was found in the aforementioned trials. As aforementioned, none of our IOI patients was naïve, and injections were given at an interval ≥ 8 weeks.

The patient that had the RO only sought medical help at 47 days after the last injection, neglecting his own early symptoms. This happened despite the advice given about the drug’s potential side effects. Sometimes some patients neglect the early symptoms for a variety of reasons, but late care probably relates to dreadful outcomes. We must bear in mind that despite all care, there will be always someone ‘out of care’ and someone ‘lost to follow up’. That is why we decided to cease the use of brolucizumab in our department in 2023. In this particular case, one may argue about why the IVI of dexamethasone was delayed by 4 days. There was a choice between administering systemic steroids promptly while waiting for the microbiology test results and giving the IVI of dexamethasone after ruling out an infectious retinitis.

This work has the limitations of being an institutional retrospective report and comprising a small sample size. Considering strengths, first and foremost, our data are not likely to overestimate the IOI events related to brolucizumab use, but rather to underestimate them. This means that the risk indicated by the OR of brolucizumab IOI events, which we conclude is greater than the risk of septic endophthalmitis related to IVI, may actually be even greater. This study describes the real-world experience of a center using brolucizumab for 3 years until the decision to suspend its use, and the first case of an RV and RO occurring in Portugal. It does not find a female predominance in brolucizumab’s IOI events, unlike what has been reported earlier. This work is confirmatory of previous reports on brolucizumab’s safety, as aforementioned, and it determined the OR of IOI with brolucizumab compared with the serious complication of septic endophthalmitis after IVI to give a clearer picture on the importance of safety while using IVI of brolucizumab.

In conclusion, there is a non-negligible safety risk of using brolucizumab as the treatment for nAMD, and the first case of IOI may occur after several eyes have been treated without complications. The risk of IOI and severe sight-threatening complications related to brolucizumab is greater than the risk of septic endophthalmitis after IVI.

## Figures and Tables

**Table 1:** Intraocular inflammation with brolucizumab.

	Age (yo)	Gender	Eye	Date of Onset of Symptoms (Month.Year)	Duration of Symptoms until Assistance	Elapsed Time since the Injection	Findings at Presentation	BCVA at Onset of Symptoms (and Letter Loss)	Number of BRO Injection	Therapy	Time to Discharge	Final BCVA	Disease
Patient 1	68	Female	RE	8.2022	24 days	21 days	Floatters Vitritis	46 L (−6 L ) (≈20/120)	first	Topical and systemic steroids	4 weeks	52 L (20/87)	PCV
Patient 2	84	Female	RE	5.2022	07 days	07 days	Floatters Keratic precipitates Tyndall 1+	70 L (+4 L) (20/40)	third	Topical steroids	2 weeks	70 L (20/40)	nAMD
Patient 3	62	Male	RE	11.2022	immediate	21 days	Keratic precipitates Tyndall 1+ Mild vitritis	73 L (−12 L) (≈20/35)	first	Subtenon triamcinolone Topical and systemic steroids	4 weeks	85 L (20/20)	PCV
Patient 4	70	Male	LE	3.2023	47 days	7 days	Pain Redness Hyperemia and ciliary congestion Vasculitis Arterial occlusion Retinal and macular ischemia	CF (−72 L)	second	Dexametasone implant Topical and systemic steroids	9 weeks	32 L (≈20/ 230)	PCV

BCVA; best corrected visual acuity, BRO; brolucizumab, L; Early Treatment Diabetic Retinopathy Study Group (ETDRS) letters, RE; right eye, LE; Left eye, CF; counting fingers, PCV; polypoidal choroidal vasculopathy, nAMD; Neovascular age-related macular degeneration; yo; years old. BCVA is expressed in ETDRS letters from the ETDRS letter chart scale and between brackets in Snellen’s.

**Table 2:** Comparison between the Proportion of IOI with Each Anti-VEGF Agent and the General Incidence of Endophthalmitis after IVI.

Condition	Events	Observations	General Incidence (%)	*P*
A (aflibercept)	2	2884	0.00048	0.403
B (brolucizumab)	4	468	0.00048	<0.001

VEGF = vascular endothelial growth factor.

**Table 3:** Determination of the Odds Ratio for IOI after IVI for each Anti-VEGF agent.

Condition A								
N	6250	12,000	18,000	24,000	30,000	36,000	42,000	48,000
p	0.598	0.635	0.643	0.647	0.638	0.642	0.645	0.646
OR	2.33	1.58	1.50	1.46	1.56	1.53	1.51	1.49
Condition B								
N	10,500	20,000	30,000	40,000	50,000	60,000	70,000	80,000
p	<0.001	0.001	<0.001	<0.001	<0.001	<0.001	<0.001	<0.001
OR	86.20	26.03	25.45	22.71	21.33	20.51	19.96	20.15

Condition A = aflibercept; Condition B = brolucizumab; Event = IOI after IVI; N = number of injections; OR = odds ratio; P (E) = probability of the event E (general incidence of endophthalmitis after IVI) to happen.

## Data Availability

Data included in this study are available in clinical files and in the national platform Retina.pt. As the access is protected by passwords, they may be provided upon reasonable request.

## Supplementary Materials

The following supporting information can be downloaded at: https://www.mdpi.com/article/10.3390/jcm13175208/s1, File S1: Statistical calculations on broluzizumab’s side effects.
