# Supplementary material for: Inflammation and Vasculitis Related to Brolucizumab
Source: J Clin Med. 2024 Sep 2;13(17):5208. doi: 10.3390/jcm13175208 (PMC11396242; doi:10.3390/jcm13175208)
Supplement: Supplementary file 1 [file jcm-13-05208-s001.zip › jcm-3131241-supplementary.pdf]

Problem, António Campos (AC):

Supposing that the incidence of an event is 0.048% (ex. endophthalmitis per intravitreal injection).

If a drug A (aflibercept) is associated with (cases/injections) 2/2884 (0.069%)
If a drug B (brolucizumab) is associated with (cases/injections) 4/468 (0.855%)

- is it possible to calculate whether a statistically significant difference exists?
- is it possible to calculate the odds ratio?

Answer (FC):

To answer I need to put the problem in a more formal way:

- let $P(E)$ = 0.00048 the probability of the event, E, to happen

- let $P(E|A)$ = 0.00069 the probability of the event, E, to happen under condition A; let ustake this probability in the sense of frequency expressed by 2/2884.

- let $P(E|B)$ = 0.00855 the probability of the event, E, to happen under condition B; let ustake this probability in the sense of frequency expressed by 4/468.

- The conditions A, and C are independent.

The answer to the first question is yes. It is possible to determine if there is a statistically significant difference between the conditions. We can determine if the proportion obtained witheach one of the conditions is different from the general incidence of the event. We must make a binomial test of a sample, using the number of cases where the event took place and the number of total cases, comparing thereafter with the general proportion of 0.00048. The statistical platform R v3.3.2 was used as the mean to this end. These are the results of the bilateral test:

condition	events	observations	General incidence (%)	*P*
A	2	2884	0.00048	0.403
B	4	468	0.00048	<0.001

It is also possible to test if there are differences between the conditions, using a Fisher's test.

conditions	*P*
A – B	0.004

As for the second question, that is, whether it is possible to calculate the relative risk or odds- ratio, the immediate answer is no. However, we can go further on the subject. It could be possible to calculate the odds ratio when comparing the conditions in themselves, but that would be pointless, since no statistically significant difference has been found. We do not have data regarding the general incidence for each one of the conditions, therefore we cannot calculate whether their incidence is higher than expected. Let us then go other way, building a table for condition A as follows:

		Event		
		Yes	No	
Condition A	Yes			
	No			

With the available data we can fill the table's first line.

		Event		
		Yes	No	
Condition A	Yes	3	2882	2884
	No			

To fill the second line we would need missing data, but let us admit that the total number ofobservations is N,

		Event		
		Yes	No	
Condition A	Yes	2	2882	2884
	No			
				N

As the probability of occurrence of the event (E) is known, it is possible to find the totalmarginal for the first column, that is

		Event		
		Yes	No	
Condition A	Yes	2	2882	2884
	No			
		$N \times P(E)$		N

Thereafter, it is possible to calculate all the other cells

		Yes	No	
Condition A	Yes	2	2882	2884
	No	$N \times P(E) - 2$	$N \times [1 - P(E)] - 2882$	N-2884
		$N \times P(E)$	$N \times [1 - P(E)]$	N

It makes then possible to calculate the odds-ratio (OR), following the expression:

$$OR = \frac{2 \times [N \times [1 - (E)] - 2882]}{2882 \times N \times (E)}$$

We may now vary the N and calculate the odds-ratio between the 2 conditions when the numberof injections increases to infinite. As the minimum value of a cell is 1, we can determine what would be the minimum value of N that would fit the conditions of the problem. As it is, the expression is applicable

$N \times (E) - 2 = 1 \Leftrightarrow N \approx 6250$ (injections), for condition A

$N \times (E) - 4 = 1 \Leftrightarrow N \approx 10500$ (injections), for condition B

The following tables show the values for the odds-ratio, to increasing numbers of N above the minimum number calculated to respect a statistical significance of $\alpha = 0.05$. The huge numbersof injections required explain why prospective works are not suitable to study this event. However, we can calculate, taking into account the relative incidence for each one of the drugsfound in the study under consideration and the general (expected) incidence of endophthalmitis, if there is an increased risk associated with conditions (drugs) A and B.

Condition A

N	6250	12000	18000	24000	30000	36000	42000	48000
p	0.598	0.635	0.643	0.647	0.638	0.642	0.645	0.646
OR	2.33	1.58	1.50	1.46	1.56	1.53	1.51	1.49

Condition B

N	10500	20000	30000	40000	50000	60000	70000	80000
p	<0.001	0.001	<0.001	<0.001	<0.001	<0.001	<0.001	<0.001
OR	86.20	26.03	25.45	22.71	21.33	20.51	19.96	20.15

It is of note that the values of the odds-ratio decrease with the increase of N, but the nature of such variation suggests the existence of an asymptote for which the true values of the odds- ratio run to, given the initial conditions of the problem.

Condition B with RV/RO: 1 event in 468 IVI

N	80000
p	0.186
OR	4.99
